# Assessment of Vascular Endothelial Growth Factor in Fresh versus Frozen Platelet Rich Plasma

**DOI:** 10.1155/2015/706903

**Published:** 2015-08-02

**Authors:** Nada Hosny, Fikry Goubran, Basma BadrEldin Hasan, Noha Kamel

**Affiliations:** Clinical Pathology Department, Faculty of Medicine, Suez Canal University, Ismailia 41522, Egypt

## Abstract

Platelet rich plasma (PRP) is hemoconcentration with platelets concentration above baseline values and high concentration of many growth factors. The aim of this study was to assess freezing effect on vascular endothelial growth factor (VEGF) release from PRP using two different activation methods to simplify its use in different clinical applications. PRP was prepared using two-centrifugation steps method from 12 qualified blood donors. VEGF concentrations were measured in fresh PRP and after freezing/thawing for one and three weeks with two methods of activation using (i) calcium gluconate and (ii) calcium gluconate and thrombin. Platelets count was significantly increased compared to baseline whole blood values in all fresh and frozen PRP samples (*p* value was <0.05). No significant difference was found between VEGF concentrations after activating fresh and frozen-thawed PRP samples for one and three weeks by calcium alone or calcium with thrombin, and also no significant difference was found when freezing period was extended from one to three weeks. Our results showed that platelets count does not correlate with variable levels of VEGF. PRP could be prepared once and preserved frozen for at least three weeks for the next treatment sessions and activation with thrombin addition to calcium will not augment the growth factor release.

## 1. Introduction

Platelet rich plasma (PRP) is a generic term referring to any sample of autologous or allogeneic plasma with platelets concentrations above baseline blood values. PRP has been known as a powerful adhesive and hemostatic agent since the 1970s and as a rich source of autologous growth factors since the 1990s [1, 2].

The use of blood components for nontransfusional purposes started in 1998 [3]. Platelet concentrate component has gained a central role for nontransfusional use. As it is a source of growth factors, it can be used, in both liquid and gel forms, to promote the regeneration of damaged tissues [4].

Being an autologous product, it is an attractive therapy due to the theoretical reduced risks and side effects compared to those commonly approved traditional commercial drugs [5].

Platelets contain growth factors in their alpha granules, such as transforming growth factor-beta (TGF-*β*), fibroblast growth factor-2 (FGF-2), platelet-derived growth factors (PDGF-AB), insulin-like growth factor-1 (IGF-1), epidermal growth factor (EGF), hepatocyte growth factor (HGF), and vascular endothelial growth factor (VEGF). These factors are thought to produce beneficial effects on the healing process in soft and hard tissues and are the most applicable way to administer a biological stimulus to several damaged tissues that might benefit from this treatment option [6].

The ultimate goal of PRP treatment is to concentrate these growth factors and reintroduce them to the site of injury [6]. Numerous types of platelet products, such as platelet rich plasma, platelet-leukocyte gel, platelet rich fibrin, and platelet gel, derived from autologous or allogeneic peripheral blood, have been used for tissue repair [7].

The effect of platelet concentrates on stimulating the regeneration of bone and soft tissues led to these blood components being used in other clinical settings, in particular in maxillofacial surgery, in dentistry (dental implants, sinus augmentation, and cleft palate), in orthopaedics and traumatology (soft tissues lesions, nonunion, and loss of bone substance following trauma or excision of cysts), in ophthalmology (lesions to the corneal epithelium), in heart surgery (sternal wound dehiscence), and in other specialities following numerous reports which suggested that the product is effective, is easy to use, and does not cause adverse reactions [4]. There is no evidence of standardization of PRP preparation and its use [6]. The most argued aspects that led to a different effect on target tissues are platelets count, methods of preparation (single- or double-centrifugation, speed, timing, and temperature), activation methods (calcium chloride/gluconate; calcium chloride/gluconate and human thrombin; calcium chloride/gluconate and batroxobin; or calcium chloride/gluconate and centrifugation) [4], leukocytes/red blood cells content, and variable therapeutic protocols (amount applied, number of injections, and intervals between injections) [5].

PRP storage is also a hot topic as the freezing/thawing allows more applicable patient management, but it is thought to impair platelet function, alter the growth factor release pattern, favor the accumulation of pyrogenic cytokines, and increase the risk of bacterial proliferation [5].

For these reasons, fresh administration of PRP is preferred (thus necessitating blood collection for each injection, in case of multiple treatment sessions) [8]. Since fresh platelets are valid for only 5 days, they are difficult to be used in therapeutic uses for their regenerative ability by outpatient doctors in different indications, which need repeated sessions. Therefore, this study aimed to assess VEGF concentration in both fresh PRP and −40°C stored PRP. In addition, we investigated a possible correlation between the platelet count and VEGF concentration.

## 2. Materials and Methods

The study is a comparative cross-sectional analytical study.

Twelve healthy subjects (10 males and 2 females, their age ranging from 21 to 37 years) who met allogeneic blood donor requirements (according to AABB guidelines, 2011) were enrolled in this study, after giving informed consents. The Institutional Research Review Board of Faculty of Medicine, Suez Canal University, approved the study protocol and the consent forms.

Donors who had infections and used nonsteroidal anti-inflammatory drug within 5 days prior to blood donation, with haemoglobin values ≤12 g/dL, or with platelet values ≤150 × 10^3^/*μ*L were excluded.

### 2.1. PRP Preparation

Each subject donated approximately 450 mL of whole blood into triple blood bags used for platelets storage for 5 days containing 63 mL CPDA-1, purchased from JMS (Ang Mo Kio, Singapore), using routine whole blood donation procedures. These bags are used routinely at our blood bank centre.

Platelet concentrates were prepared from the donated whole blood within two hours of collection. The whole blood was centrifuged at 750 g for 7 minutes (start to finish), with 5 minutes of brake time to prepare PRP which was then transferred to a satellite bag. Then, PRP was centrifuged at 5,300 g for 10 minutes to prepare a platelet concentrate (centrifuge model: RC 12BP plus, Thermo scientific Inc., Waltham, USA). Leukoreduction of platelet concentrates was not done. Most of the plasma was transferred to another satellite bag. An average of 50 ± 5 mL of plasma was left with the platelet concentrate.

### 2.2. Thrombin Preparation

Ten mL of venous blood was collected in plain Vacutainer tubes from each donor, was put aside to clot, and was centrifuged at 3,000 rpm for 10 minutes. Serum was separated.

### 2.3. Evaluation of Platelet Count and VEGF Released from PRP

Platelets counts were assessed in whole blood and PRP (fresh and frozen) using fully automated hematology analyzer (Horiba ABX Micros 60, France). Platelets count concentration factor (platelet yield) was calculated by dividing platelets count of PRP by platelets count of whole blood.

One hour after separation, 14 mL of PRP was collected from each PRP unit after gentle mixing by inversion and was aliquoted into three tubes. One tube was assayed immediately for baseline VEGF concentration, while the other two aliquots were kept frozen in −40°C. One of the aliquots was stored for one week, and the other for three weeks.

For each of the three aliquots, PRP was divided into two parts and activated shortly before VEGF assay by either calcium gluconate or “calcium gluconate and thrombin” as follows:

 two mL of PRP + 300 *μ*L of calcium gluconate (0.05 gm/mL);
 two mL PRP + 500 *μ*L of thrombin + 300 *μ*L of calcium gluconate (0.05 gm/mL).

All activated PRP samples (fresh and frozen/thawed) were incubated for 30 minutes at room temperature, without another centrifugation to get releasate for VEGF assessment.

VEGF was measured by commercially available Quantikine ELISA kits (R&D Systems, Minneapolis, USA), according to manufacturer's instructions.

### 2.4. Statistical Analysis

Data were analysed using SPSS software (SPSS Inc. version 17, Chicago, USA). Data were presented as mean ± standard deviation (SD).

Student's *t*-test was used to compare between fresh and frozen PRP samples after activation by calcium and calcium and thrombin. Regression analysis (Spearman rank correlation coefficient) was done to correlate between PRP platelet count and VEGF concentrations. Significant difference was defined as *p* < 0.05.

## 3. Results

The mean initial platelet count in whole blood (*n* = 12) was 282 ± 56 × 10^3^/*μ*L. It was significantly increased in fresh PRP after separation to reach 991.4 ± 378 × 10^3^/*μ*L.

RBCs and WBCs were remarkably depleted in PRP compared with the initial concentrations in whole blood samples (Table 1).

Platelet concentrations significantly increased after double-spin PRP preparation where mean platelet yield in fresh PRP was 3.5. The platelet yield decreased after freezing PRP samples; yet it was still significant when compared to the initial whole blood platelets concentration (*p* value < 0.05) (Table 2).

A baseline measurement of VEGF concentration was done in each fresh PRP sample; its mean ± SD was 160.8 ± 88 pg/mL.

VEGF levels significantly increased after activating fresh and frozen/thawed PRP samples for one and three weeks with either calcium or calcium/thrombin. VEGF concentrations in calcium activated PRP samples significantly increased from 160.8 ± 88 to 545.2 ± 349 pg/mL (fresh plasma), 339.5 ± 190 pg/mL (after one week of freezing/thawing), and 339.9 ± 189 pg/mL (after three weeks of freezing/thawing) (*p* value < 0.05 each). Regarding the addition of thrombin to calcium as an activator, VEGF concentrations significantly increased in all PRP samples (fresh, one-week frozen, and three-week frozen) (*p* value < 0.05); they were 608.7 ± 494, 365.2 ± 189, and 365.6 ± 188 pg/mL, respectively.

VEGF in calcium activated fresh PRP samples was nonsignificantly higher than that in frozen/thawed PRP samples. In addition, there was nonsignificant difference in VEGF results in frozen samples for one or three weeks. The same results were revealed in VEGF concentration in calcium and thrombin activated PRP samples.

The mean values of VEGF concentration in calcium activated PRP samples are nonsignificantly lower (*p* value > 0.05) than those activated by calcium and thrombin in all types of samples (fresh, one-week freezing/thawing, and three-week freezing/thawing) (Table 3).

**Figure 1:**
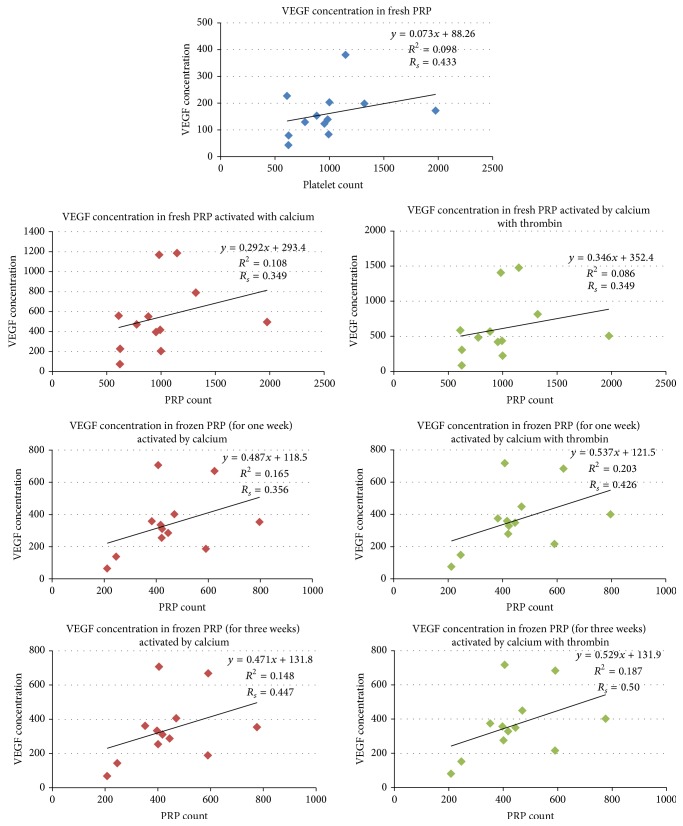
Correlations between VEGF concentrations and platelets count in different PRP activation and storage periods. *R*_*s*_, Spearman rank correlation coefficient.

Using the Spearman rank correlation coefficient, variation in VEGF levels did not significantly correlate with variations in PRP platelets count (they showed only fair degree of linear relationship where *R*_*s*_ ranged from 0.34 to 0.50) (Figure 1).

## 4. Discussion

During the past two decades, numerous researches have been published in an attempt to characterize and classify the several techniques for PRP preparation (centrifugation speed and the used anticoagulant), content (leucocytes and growth factors), and applications with no consensus [9]. There is increasing awareness on the need for PRP standardization starting from their preparation up to their clinical application. Among the various factors that need more investigations is the possibility to store PRP. As some researchers avoid freezing/thawing, fearing of potential deleterious effects on platelet function and growth factor release pattern [5].

In this regard, our aim was to test whether PRP can be used either as fresh or frozen/thawed, in different clinical applications that need repetitive sessions with preserved VEGF content. Thus, we measured the level of VEGF in fresh and frozen/thawed PRP and investigated the activation with calcium alone compared to calcium and thrombin.

We used a two-centrifugation-step method; the first step was to deplete the product of red and white blood cells with minimal loss of platelets, and the second step was to obtain the highest recovery and the best yield of platelets in the smallest final plasma volume [10].

Platelets purity was monitored in PRP by quantifying other cell types. RBCs and WBCs were remarkably depleted in PRP and remained at a very low concentration compared with the initial mean concentrations for both cell types. Similar results were reported by Amable et al. [10], who observed that initial RBCs and WBCs concentrations were remarkably depleted and remained at very low concentrations (<0.24% for RBCs and <0.28% for WBCs) after PRP preparation.

There is a paucity of data in assessing frozen/thawed PRP platelet counts and growth factors release; our results revealed that the count decreased almost to half the baseline PRP platelets count for the one-week storage samples at −40°C and did not change when the storage period was extended to three weeks. This decrease in count may be due to effect of either time or temperature or both.

Our results also showed that VEGF concentrations in fresh PRP showed nonsignificantly higher values than in frozen/thawed PRP for one or three weeks at −40°C when calcium alone was used as an activator, while results after freezing for one and three weeks did not significantly change. Furthermore, the same results were detected regarding values of calcium and thrombin activated PRP (*p* > 0.05).

Recently, Roffi and colleagues [11], who investigated whether PRP freezing/thawing affected the release of growth factors from platelets alpha-granules at two time points, obtained similar results, 1 hour (immediate release) and 7 days, as scheduled delivery time in the clinical application. No significant differences were detected for VEGF levels after freezing at one hour and at 7 days. They concluded that PRP cryopreservation is a safe procedure, which sufficiently preserves PRP quality and its biological activity.

Thus, freezing/thawing might still be a valid option to store PRP, although in this case frozen PRP might be less sensitive than fresh PRP to calcium, by not liberating the total amount of growth factors stored in the alpha-granules, and some platelets and bioactive molecules might be damaged [11].

We found that the mean VEGF concentrations in “calcium and thrombin” activated PRP showed no significant difference compared to the mean concentrations in calcium activated PRP, in either fresh or frozen/thawed PRP. To our knowledge, no other studies discussed the difference in growth factors release by PRP when activated by calcium alone and after activation by calcium and thrombin in either fresh or frozen PRP.

Our results showed that VEGF concentrations did not correlate with variations in PRP platelets count. In accordance, Bausset et al. [9] had reported that VEGF levels did not correlate with variation in platelets count. Similar observations were reported by Weibrich et al. [12], indicating that platelet count may not be an appropriate indicator to predict biological activity of platelets.

Therefore, there is no simple procedure available to obtain preoperative estimation of the content of individual growth factors in PRP. This information would be helpful to ensure a reliable and reproducible use of PRP for clinical treatment, since the regenerative potential of PRP undoubtedly depends on its growth factor levels [13]. Thus, the development of new strategies allowing preinjection testing of PRP biological activity is challenging, as there is a high interindividual variability in cellular production and storage of cytokines [9].

The actual challenge for PRP optimization is to determine the main bioactive components responsible for the clinical effects. Not the platelets count nor the platelet growth factors content but the synergy of both should be considered.

The limitation of this study is that freezing time was prolonged to only three weeks.

## 5. Conclusion

This study underlines two controversial issues. (1) PRP freezing/thawing does not significantly affect the release of VEGF. (2) The different release kinetics is not significantly influenced by the two investigated activating agents. However, until clinical studies explore and clarify the effects of PRP storage on patient symptoms and functional improvement, this study suggests that freezing/thawing does not significantly affect PRP and can be considered as a storage option and thus simplify the management of patients undergoing multiple injection sessions of PRP.

## Figures and Tables

**Table 1:** Red blood cell and white blood cell counts in whole blood and fresh PRP.

	RBCs (×10^6^/*μ*L)	WBCs (×10^3^/*μ*L)
	(mean ± SD)	(mean ± SD)
Whole blood	4.1 ± 0.3	7.7 ± 2.3
Fresh PRP	0.02 ± 0.01	0.1 ± 0.02

RBCs: red blood cells; WBCs: white blood cells.

**Table 2:** Platelet yield of fresh and one-week and three-week frozen/thawed PRP.

	Platelet count ×10^3^/*μ*L	Platelet yield	*p* value
	(mean ± SD)	(mean ± SD)	
Whole blood	282 ± 56		
Fresh PRP	991.4 ± 378	3.5 ± 0.9	<0.001^*∗*^
Frozen PRP (1 week)	453.2 ± 158	1.7 ± 0.6	<0.001^*∗*^
Frozen PRP (3 weeks)	441.6 ± 154	1.6 ± 0.6	0.001^*∗*^

^**∗**^Statistically significant difference (*p* < 0.05).

**Table 3:** VEGF concentration (pg/mL) in calcium versus calcium and thrombin activated fresh and frozen/thawed PRP samples.

	Ca activated PRP VEGF concentration	Ca and thrombin activated PRP VEGF concentration	*p* value
Fresh	545.2 ± 349	608.7 ± 494	0.695 (NS)
Frozen/thawed (1 week)	339.5 ± 190	365.2 ± 189	0.743 (NS)
Frozen/thawed (3 weeks)	339.9 ± 189	365.6 ± 188	0.741 (NS)

NS: not significant.
